# Microstructure and Constitutive Equation of Hot Compressive Fe-15Mn-15Al-5Ni-1C Low-Density Steel

**DOI:** 10.3390/ma15082721

**Published:** 2022-04-07

**Authors:** Yingjie Wang, Fengya Hu, Zhongjun Wang, Kuijun Fu, Weijuan Li, Jiaji Wang, Jing Guo

**Affiliations:** 1 School of Materials and Metallurgy, University of Science and Technology Liaoning, Anshan 114051, China; ustlyingjiewang@126.com (Y.W.); ustllwj@gmail.com (W.L.); guojing_neu@163.com (J.G.); 2 State Key Laboratory of Metal Material for Marine Equipment and Application, Ansteel Group Corporation, Anshan 114009, China; agfkj63@gmail.com (K.F.); agwjj2013@gmail.com (J.W.)

**Keywords:** low-density steel, as-cast state, DRX, microstructure, constitutive equation, κ carbide

## Abstract

The hot deformation behavior and dynamic recrystallization (DRX) of Fe-15Mn-15Al-5Ni-1C low-density steel in the as-cast state was investigated via hot compression experiments over temperature and strain rate ranges of 925 to 1150 °C and 0.01 to 10 s^−1^, respectively. A constitutive equation and a critical DRX model of the Fe-15Mn-15Al-5Ni-1C low-density steel were also constructed. The results showed that higher strain rates resulted in significant work hardening and subsequent rapid softening of the Fe-15Mn-15Al-5Ni-1C low-density steel, while lower strain rates resulted in predominantly steady-state flow behavior. The activation energy of deformation for the Fe-15Mn-15Al-5Ni-1C low-density steel was Q = 540 kJ mol^−1^ and the stress index was n = 4. The hot deformation mechanism was solute dragging and dislocation climbing, which was controlled by the strain rate. Increasing the deformation temperature or strain rate reduced the critical stress value σ_c_ of the DRX of the Fe-15Mn-15Al-5Ni-1C low-density steel and contributed to the DRX of austenite and δ-ferrite. The Fe-15Mn-15Al-5Ni-1C low-density steel after the hot compression deformation was mainly composed of austenite, ferrite, and κ carbide phases.

## 1. Introduction

In recent years, the automotive industry has been facing a huge challenge in terms of energy saving and emissions reduction due to the increasing problems of resource scarcity and environmental degradation [1]. The development of lightweight steel for new energy vehicles has attracted widespread academic attention. The new generation of steel for new energy vehicles should have a low density while having high strength and high toughness. The Fe-Mn-Al-C series of low-density steels consists of lightweight steels that can be widely used in automotive manufacturing [2], which exhibit excellent ductility and high strength. However, Fe-Mn-Al-C low-density steels still suffer from processing problems, such as segregation [3]. Therefore, it is important to study the hot deformation of Fe-Mn-Al-C low-density steels with different alloying elements added in order to optimize the TMP (thermomechanical processing) and improve the steel properties. In previous studies, Ley et al. [4] found the presence of κ-type carbides, which had face-centered cubic structures with the chemical formula (Fe, Mn)_3_AlC and a precipitate during aging treatment at 750 K. This promoted work hardening of the alloy, which resulted in strengthening. Wan et al. [5] studied Fe-Al-Mn-C low-density steel and found that the alloy was negatively sensitive to temperature and positively sensitive to the strain rate, and the flow stress increased with increasing strain rate or decreasing deformation temperature. Mozumder et al. [6] conducted hot compression experiments on Fe-9Al-10.8Mn-4.5Ni-0.7C low-density steel at temperatures and strain rates ranging from 950 to 1150 °C and 0.001 to 10 s^−1^, respectively, and obtained an average hot activation energy of 400 kJ mol^−1^ for this low-density steel. The coexistence of austenite and δ-F made the hot deformation more complex.

Dynamic recrystallization (DRX) is a fundamental feature of microstructures in controlling grain sizes and mechanical properties during hot deformation [7], which indicates that DRX studies on low-density steels are essential. Liu et al. [8] conducted hot compression experiments on Fe-11Mn-10Al-0.9C low-density steel to investigate the DRX and dynamic precipitation behavior of κ-type carbides in low-density steel and found that the coupling effect of the dynamic precipitation–transformation–recrystallization of κ-type carbides during hot treatment was beneficial in regulating the microstructure. However, in previous studies on the thermal deformation behavior of low-density steels of the Fe-Mn-Al-C series, the object of study has often been the rolled low-density steel, while less research has been reported on cast low-density steel, considering that the hot deformation study on the cast steel has more reference significance for the actual production; therefore, the object of study in this research was the cast low-density steel.

Compared to Fe-Mn-C steels, Fe-Mn-Al-C low-density steels with added aluminum have lower densities, decreasing by 1.3% for every 1 wt.% of Al added [9], while offering properties such as higher yield strengths and elongation, lower susceptibility to solidification cracking, and higher corrosion resistance [10]. In previous studies on low-density steels of the Fe-Mn-Al-C family, the hot deformation behavior of Fe-Mn-Al-C steels with an Al content of less than 10 wt.% was studied in depth, but there is a lack of research on low-density steels with an Al content above 10 wt.%. Ni can expand the austenite zone and is the main alloying element for the formation and stabilization of austenite. Therefore, Ni can improve the plasticity and toughness of low-density steels to some extent. Kim et al. [11] found that Ni could promote the precipitation strengthening of the B2 phase, greatly improving the strength and ductility of the steel. Although the addition of a higher content of Al will reduce the density of low-density steel, it will also reduce the plasticity of low-density steel; therefore, in order to both reduce the density of steel and maintain the high strength and high plasticity of low-density steel, this study added a moderate amount of Ni under the premise of adding Mn, C, and Al.

In this experiment, an Fe-15Mn-15Al-5Ni-1C low-density steel was developed to investigate its flow curve characteristics. We constructed a flow stress constitutive model and a DRX critical model, as well as constraining the relationship between the steel’s DRX microstructure and deformation conditions through hot compression experiments. The constitutive equation, which was constructed using hyperbolic sine functions, had good predictive effects and provided a theoretical reference for TMP. It is worth noting that current research on the constitutive equation and recrystallization prediction model of as-cast low-density steel with Ni addition is limited; therefore, our work provided research implications for the development of low-density steels.

## 2. Materials and Methods

**Figure 1:**
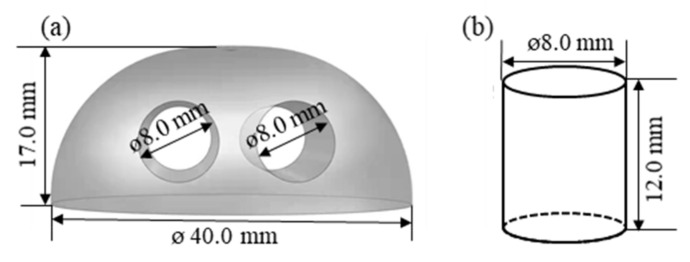
(**a**) Low-density steel cast ingot used for experiment; (**b**) cylindrical hot compression specimen.

**Figure 2:**
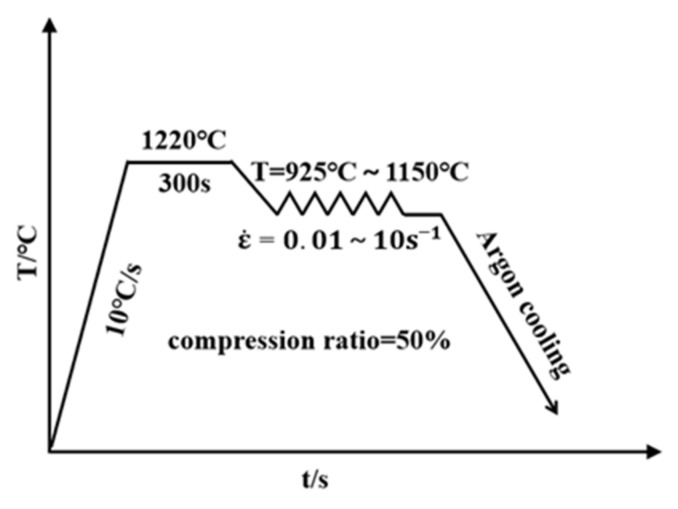
Schematic illustration of the hot compression scheme for the Fe-15Mn-15Al-5Ni-1C low-density steel.

The experimental material was Fe-15Mn-15Al-5Ni-1C low-density steel cast ingots, which were refined with the help of the Shenyang Institute of Metal Research and whose chemical composition is shown in Table 1. A cylindrical specimen of Φ8.0 mm × 12.0 mm was taken from the cast ingot via a wire cutting technique as the experimental sample, the size of the cast ingot, and the location of the sample, as shown in Figure 1. Hot compression experiments were carried out on a THERMECMASTOR 100 KN hot simulation experimental machine and the experimental protocol is shown in Figure 2. The samples were heated to 1220 °C at a heating rate of 10 °C s^−1^ and kept for 5 min, then cooled down to each hot deformation temperature at a cooling rate of 5 °C s^−1^ for isothermal deformation. Due to the limited experimental material and in order to make a slightly larger range of application of the intrinsic constitutive equations, the study was not carried out using equally spaced temperatures. The hot compression temperatures were 925 °C, 1000 °C, 1100 °C, and 1150 °C, and the strain rates were 0.01 s^−1^, 0.1 s^−1^, 1.0 s^−1^, and 10 s^−1^, all at a strain value of 0.7. The samples were cooled using argon immediately after the deformation was completed to retain the post-deformation microstructure. The samples were then cut symmetrically along the axial direction after the hot compression experiment to obtain experimental samples before being subjected to the metallographic experiment. The experimental specimens were ground, polished, and then tint etching was accomplished using a normal 4% Nital solution, followed by quick immersion in Villela’s reagent (100 mL ethanol, 5 mL HCl, and 1 g dry picric acid). The microstructure was observed under an Axioscope 2 MAT optical microscope (Zeiss, Thuringia, Germany) after cleaning and blow drying, and the fine structure was observed using a Zeiss-Ultra-55 field emission scanning electron microscope (SEM, Thuringia, Germany) equipped with an energy dispersive spectrometer (EDS). An X’Pert Powder X-ray diffractometer (XRD) was also used for the physical phase analysis of the experimental specimens.

## 3. Results and Discussion

### 3.1. As-Cast Microstructure

**Figure 3:**
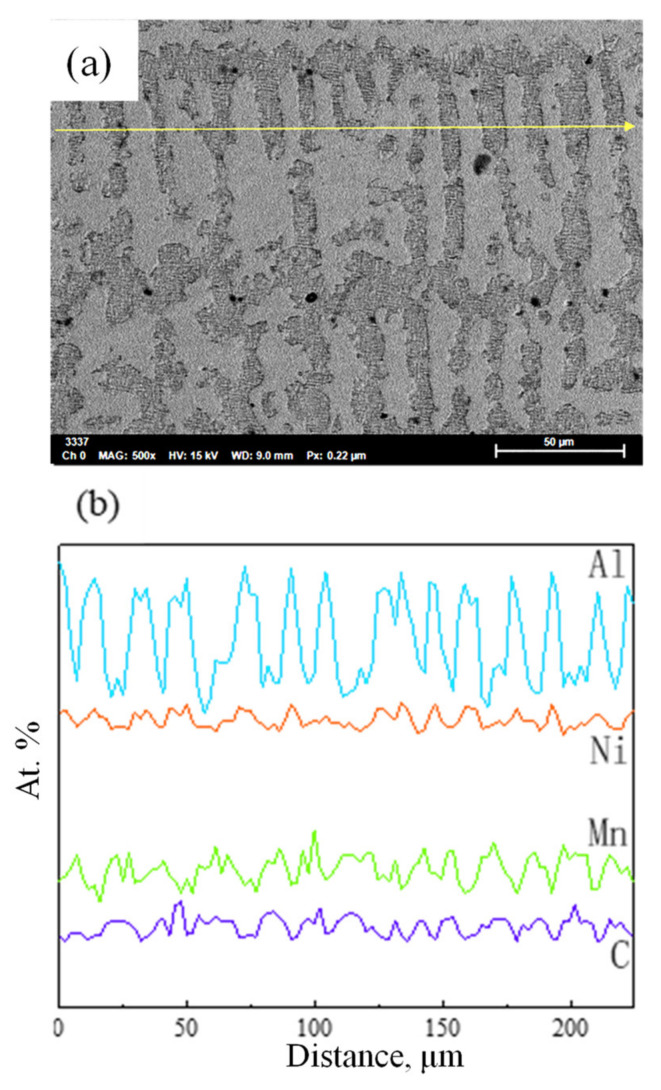
(**a**) As-cast microstructure of the Fe-15Mn-15Al-5Ni-1C low-density steel; (**b**) line scan results for the elements Al, Ni, Mn, and C.

The as-cast microstructure of the Fe-15Mn-15Al-5Ni-1C low-density steel and the line scan results of the four elements Al, Ni, Mn, and C, are shown in Figure 3. Two types of coarse dendrite microstructures were observed in Figure 3a: austenitic (A) dendrites and high-temperature ferritic (δ-F) dendrites. The δ-F dendrites were well developed and more branched, which was attributed to the fact that while the columnar crystals grew, the liquid metal at the center of the ingot met the demand of the nuclei for supercooling such that the hot dissipation process was no longer selective in any directions, thus enabling the nuclei in the liquid phase to grow uniformly in all directions. This allowed the nuclei in the liquid phase to grow uniformly in all directions, eventually promoting the formation of equiaxed crystals. Equiaxed crystals that were closer together interacted with each other during the growth process, and the branches overlapped each other, eventually forming dendritic crystals. The differences in the distribution of the elements between the austenite and ferrite phases can be observed in Figure 3b. The distributions of the elements Al and Ni in the as-cast tissue were similar, with a higher content in the white ferrite than in the black austenite. However, the contents of Mn and C elements in ferrite were lower than in austenite. This was because the Mn and C elements were austenite forming and were less soluble in the ferrite than in austenite, thus causing the Mn and C elements to aggregate toward austenite. 

### 3.2. Flow Behavior

**Figure 4:**
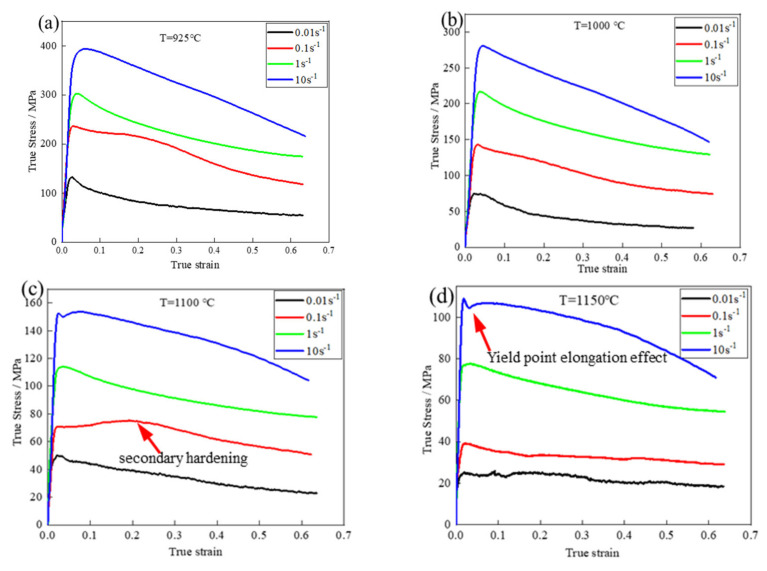
True stress and true strain behavior of hot compression experimental alloys at different temperatures: (**a**) 925 °C; (**b**) 1000 °C; (**c**) 1100 °C; (**d**) 1150 °C.

Figure 4 shows the stress–strain curves of the experimental samples of the Fe-15Mn-15Al-5Ni-1C low-density steel under different deformation conditions. The flow stress curves were divided into three stages. The first was the early stage of deformation, where the strain increased proportionally to the stress as the load increased and the material underwent elastic deformation such that the stress–strain curve is straight. In the second stage, the material was deformed plastically as the strain continued to increase, where the Fe-15Mn-15Al-5Ni-1C low-density steel had a work hardening effect, at which time, the recrystallization softening effect was not obvious and work hardening dominated, resulting in a rapid increase in stress. As the strain increased, the recrystallization softening effect gradually increased, and eventually, the work hardening effect and recrystallization softening effect reached a balance, causing the flow stress to reach a peak. In the third stage, recrystallization softening dominated after the peak stress, and as the strain continued to increase, the DRX softening effect increased and the stress started to decrease until they reached a steady state [12,13]. 

When the deformation temperature of the Fe-15Mn-15Al-5Ni-1C low-density steel was constant, the flow stress increased with increasing strain rate and the strain corresponding to the peak stress also increased. On the other hand, when the strain rate was constant, the flow stress increased with decreasing deformation temperature and the strain corresponding to the peak stress also increased. This was because the lower strain rate prolonged the time of accumulated distortion energy and dislocation annihilation, allowing the DRX grains to nucleate and grow sufficiently such that the softening effect was strong [14]. On the other hand, a higher deformation temperature increased the driving force for dislocations to cross slip and creep [15], the atoms were more reactive, and recrystallization was more likely to occur. The decrease in strain rate prolonged the time of accumulated distortion energy and dislocation annihilation, which facilitated the occurrence of softening behaviors, such as DRX. 

Unlike other steels, the Fe-15Mn-15Al-5Ni-1C low-density steel showed secondary work hardening at a hot deformation temperature of 1100 °C and a deformation rate of 0.1 s^−1^, as shown in Figure 4c, where the flow stress again showed an obvious peak. After the flow stress reached a peak, the deformation of the alloy was again dominated by the work-hardening effect with a secondary hardening effect. This was because, with an increase in strain, the load was transferred from δ-F to austenite, resulting in an increase in the dislocation density and flow stress until DRX occurred in the austenite. The flow stress slowly increased with increasing strain, reached a peak, and then decreased in a slow manner, indicating that the steel had typical DRX properties under the current deformation conditions. However, it is worth noting that the stress–strain relationship of the Fe-15Mn-15Al-5Ni-1C low-density steel was different at high temperatures and high strain rates. From Figure 4d, the stress–strain curve of the Fe-15Mn-15Al-5Ni-1C low-density steel at a hot deformation temperature of 1150 °C and deformation rate of 10 s^−1^ showed a yield point elongation effect [16], where distinct upper and lower yield points were observed. This could be explained by the non-uniform strain distribution and the DRX of δ-F [17]. Because δ-F was softer, austenite was more resistant to plastic deformation and the strain was mainly adapted to δ-F, resulting in a higher dislocation density of δ-F than austenite. In addition, the Fe-15Mn-15Al-5Ni-1C low-density steel used in this project was high in Al content, leading to a higher stacking fault energy in δ-F, which was more prone to cross slipping and DRX. When the strain at which DRX occurred in δ-F was reached, the strain energy that accumulated during hot deformation was suddenly released as the flow stress decreased, which was also responsible for the elongation of the yield point.

### 3.3. Constitutive Analysis

Under high-temperature plastic deformation circumstances, the flow stresses were impacted by both the deformation temperature and the strain rate. The hyperbolic sine model presented by Sellars [18] described the connection between flow stress, strain rate, and deformation temperature for hot deformation as follows:

(1) $$\dot{\varepsilon }=A{\left[\sin h\left(\alpha \sigma \right)\right]}^{n}\exp\left[-Q/\left(\mathrm{RT}\right)\right]$$

where $\dot{\varepsilon }$ (s^−1^) is the strain rate; A (s^−1^) and α (MPa^−1^) are material constants; σ is the flow stress, which has the unit of MPa; n is the stress index; R is the universal gas constant, which has the value 8.314 J/(mol·K); T is the thermodynamic temperature (K); and Q is the activation energy of the thermal deformation of the material, which has the unit of kJ mol^−1^.

When ασ < 0.8, Equation (1) can be simplified to Equation (2), and when ασ > 1.2, Equation (1) can be simplified to Equation (3) [19]:

(2) $$\dot{\varepsilon }=A_{1}\sigma^{n1}\exp[-Q/(\mathrm{RT})]$$

(3) $$\dot{\varepsilon }=A_{2}\exp(\beta \sigma )\exp[-Q/(\mathrm{RT})]$$

where A_1_, A_2_, and β = αn_1_ are material constants that are independent of temperature. The representative stress of each flow curve can be expressed in terms of the peak stress σ_p_ in the flow curve [20]. 

According to the applied peak stress σ_p_ obtained from the stress–strain curve, the constitutive parameters in this work were calculated and the flow characteristics of the as-cast Fe-15Mn-15Al-5Ni-1C low-density steel during the hot deformation process were studied. 

**Figure 5:**
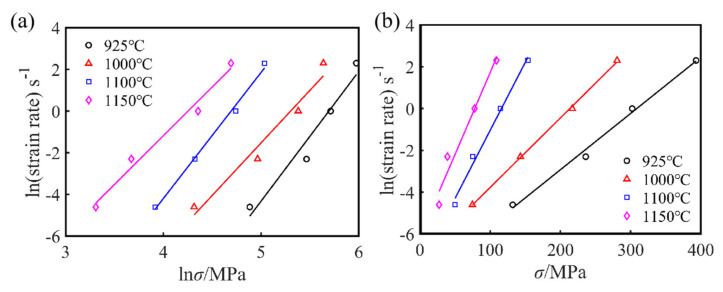
(**a**) ln$\dot{\varepsilon }$–lnσ and (**b**) ln$\dot{\varepsilon }$–σ linear regression curves at peak stress for the Fe-15Mn-15Al-5Ni-1C low-density steel.

**Figure 6:**
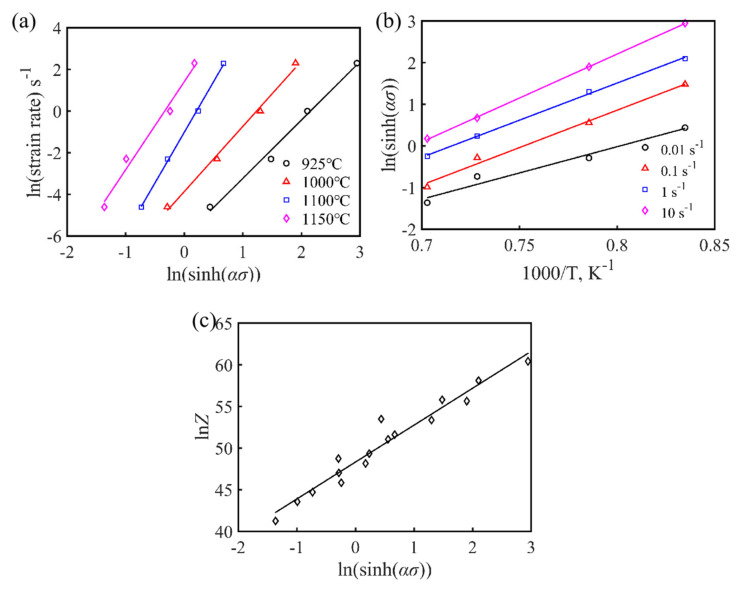
(**a**) ln$\dot{\varepsilon }$–ln[sinh(ασ)], (**b**) ln[sinh(ασ)]–1000/T, and (**c**) lnZ–ln[sinh(ασ)] linear regression relationships at peak strain for the experimental alloy.

The average slopes, n1, β, and n, of the ln$\dot{\varepsilon }$ versus lnσ, σ, and ln[sinh(ασ)] curves, respectively, were obtained by taking the natural logarithm of both sides of Equations (1)–(3) simultaneously, as shown in Figure 5 and Figure 6a. For the given deformation conditions, the values of n_1_, β, and α for the as-cast Fe-15Mn-15Al-5Ni-1C dual-phase low-density steel were 5.501, 0.05091, and 0.009254, respectively.

The expression for the hot deformation activation energy Q can be derived mathematically by applying Equation (1):

(4) $$Q=R\cdot {\left.\frac{\partial \ln\dot{\varepsilon }}{\partial \ln[\sin h(\alpha \sigma )]}\right|}_{T}\cdot {\left.\frac{\partial \ln[\sin h(\alpha \sigma )]}{\partial \left(1/T\right)}\right|}_{\dot{\varepsilon }}=\mathrm{RnH}$$

where n is the stress index, which is also the average slope of the linear-fitted curve of ln$\dot{\varepsilon }$–ln[sinh(ασ)] for a given temperature, as shown in Figure 6a, and H is the average slope of the four linear-fitted curve of ln[sinh(ασ)]–1/T for a given temperature. According to the linear-fitted results presented in Figure 5a,b, the average value of the hot deformation activation energy Q for the as-cast Fe-15Mn-15Al-5Ni-1C low-density steel under different deformation conditions was calculated as 540 kJ mol^−1^.

Many researchers [6,8,21] have studied the hot deformation behavior of Fe-Mn-Al-C low-density steels and have given the hot deformation activation energy of these materials. Table 2 shows the hot deformation activation energy of different Fe-Mn-Al-C-based low-density steels. From Table 2, it can be seen that the hot deformation activation energy value Q of each low-density steel was different due to the different alloy compositions. Compared with these low-density steels, Fe-15Mn-15Al-5Ni-1C low-density steel had a higher Q value because of the high contents of Mn, Al, Ni, and C elements in Fe-15Mn-15Al-5Ni-1C low-density steel, the Mn and Ni elements replaced the Fe atoms in γ-Fe to form a replacement solid solution, and the C atoms dissolved into δ-F to form an interstitial solid solution, resulting in enhanced solid solution strengthening. Dislocation slipping was impeded by the solute atoms, making it necessary for the atoms to have higher energy during thermal deformation for the material to deform. At the same time, Al is a ferrite-forming element and higher Al content promotes the formation of δ-F, increasing the heat deformation activation energy of Fe-15Mn-15Al-5Ni-1C low-density steels. In addition, higher Al content and C content also promote the formation of κ carbides, which impede dislocation motions via the pinning effect and increase the heat deformation activation energy. Finally, the higher elemental Al and Ni content of Fe-15Mn-15Al-5Ni-1C low-density steel increased the layer dislocation energy of Fe-15Mn-15Al-5Ni-1C low-density steel, inhibited the DRX behavior of Fe-15Mn-15Al-5Ni-1C low-density steel, and increased the heat deformation activation energy. 

Compared to Fe-11Mn-10Al-0.9C low-density steel [8] and Fe-27Mn-11.5Al-0.95C low-density steel [21], Fe-15Mn-15Al-5Ni-1C low-density steel had a higher content of κ-type carbides; therefore, Fe-15Mn-15Al-5Ni-1C low-density steel had a higher Q value than these two low-density steels. For the Fe-9Al-10.8Mn-4.5Ni-0.7C low-density steel [6], the B2 phase and the κ carbides were present in this steel, and the B2 phase also played a role in the pigging effect. This is why the Q value of this low-density steel was also higher than that of the previous two steels, but due to the higher content and larger size of the κ-type carbides in the Fe-15Mn-15Al-5Ni-1C low-density steel, as well as the presence of a higher content of δ-F, the Q value of the Fe-15Mn-15Al-5Ni-1C low-density steel was higher.

The Zener–Hollomon (Z) parameter can be used to reflect the effect of deformation temperature and strain rate on the flow behavior of Fe-15Mn-15Al-5Ni-1C low-density steel, which is derived from Equation (5):

(5) $$Z=A{\left[\sin h(\alpha \sigma )\right]}^{n}=\dot{\varepsilon }\;\exp(Q/\mathrm{RT})$$

Mathematical manipulation of Equation (5) can be used to obtain Equation (6):

(6) lnZ=lnA+nln[sinh(ασ)]

The results with the linear regression based on lnZ and ln[sinh(ασ)] are shown in Figure 6c, and the value of A corresponding to the intercept value was 3.95 × 10^2^, while the value of its slope represents the value of the stress index n of the Fe-15Mn-15Al-5Ni-1C low-density steel, where different values of n reflect different creep mechanisms. When n = 3, the main deformation mechanism of the alloy was solute dragging, while when n = 5, creep controlled by dislocation climbing was the main deformation mechanism [22]. The values of n for the as-cast Fe-15Mn-15Al-5Ni-1C low-density steel ranged from 3 to 5 under the different strain conditions, indicating that the dislocation creep mechanism of this low-density steel was controlled by both solute dragging and dislocation climbing, where dislocation climbing at high temperatures led to DRX and softening of the alloy. 

The hot deformation constitutive equation of the Fe-15Mn-15Al-5Ni-1C low-density steel can be obtained by substituting all the parameter values obtained from the above calculations into Equation (1), as shown in Equation (7). Figure 6c reflects the linear relationship between the peak stress value σ_p_ and the Z value, which indicated that the established constitutive equation was valid.

(7) $$\sigma_{p}=\frac{1}{0.009254}\ln\left\{{\left(\frac{Z}{3.95\times 10^{20}}\right)}^{\frac{1}{4.0}}{\left[{\left(\frac{Z}{3.95\times 10^{20}}\right)}^{\frac{2}{4.0}}+1\right]}^{\frac{1}{2}}\right\}$$

(8) $$Z=\dot{\varepsilon }\exp(\frac{540}{\mathrm{RT}})$$

**Figure 7:**
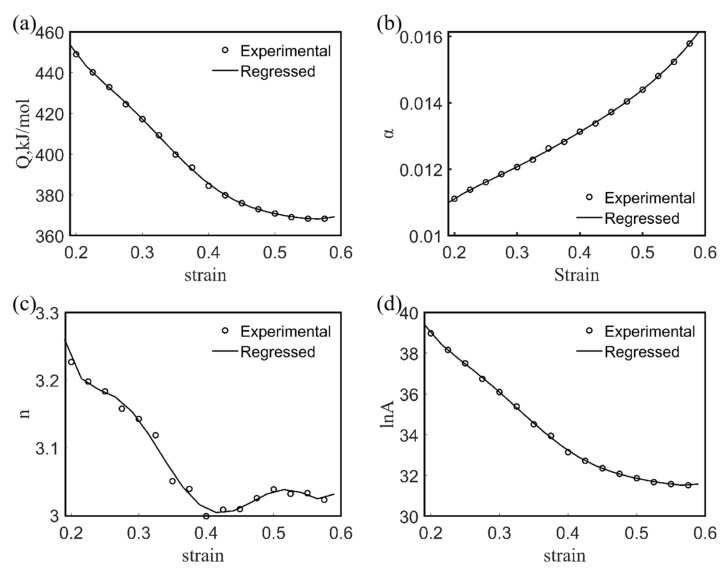
Curves of Q (**a**), α (**b**), n (**c**), and ln A (**d**) as a function of true strain for Fe-15Mn-15Al-5Ni-1C low-density steel.

Most previous studies showed that the effect of ε on the deformation activation energy and material constants was significant in a specific range [23,24]; thus, the introduction of the value of the strain ε into the constitutive equation should be considered. Equation (9) employs a sixth-order polynomial function to characterize the relationship between the α, n, Q, and lnA values of the Fe-15Mn-15Al-5Ni-1C low-density steel as functions of the value of the strain ε [25]. Figure 7 gives the results of fitting the deformation activation energy Q and the values of material constants α and A, as well as the stress index n with the value of ε. The results of the fit shown in Figure 7 are given in Table 3, which were used to determine the constitutive equations influenced by the strain variables, as described in Equation (10).

(9) $$\left\{\begin{array}{c} \alpha =X_{0}+X_{1}\varepsilon +X_{2}\varepsilon^{2}+X_{3}\varepsilon^{3}+X_{4}\varepsilon^{4}+X_{5}\varepsilon^{5}+X_{6}\varepsilon^{6}\\ n=Y_{0}+Y_{1}\varepsilon +Y_{2}\varepsilon^{2}+Y_{3}\varepsilon^{3}+Y_{4}\varepsilon^{4}+Y_{5}\varepsilon^{5}+Y_{6}\varepsilon^{6}\\ Q=Z_{0}+Z_{1}\varepsilon +Z_{2}\varepsilon^{2}+Z_{3}\varepsilon^{3}+Z_{4}\varepsilon^{4}+Z_{5}\varepsilon^{5}+Z_{6}\varepsilon^{6}\\ \ln A=E_{0}+E_{1}\varepsilon +E_{2}\varepsilon^{2}+E_{3}\varepsilon^{3}+E_{4}\varepsilon^{4}+E_{5}\varepsilon^{5}+E_{6}\varepsilon^{6} \end{array}\right.$$

(10) $$\sigma_{T,\dot{\varepsilon },\varepsilon }=\frac{1}{\alpha }\ln\left\{{\left(\frac{Z}{A}\right)}^{1/n}+{\left[{\left(\frac{Z}{A}\right)}^{2/n}+1\right]}^{1/2}\right\},\;Z=\dot{\varepsilon }\exp(\frac{Q}{\mathrm{RT}})$$

**Figure 8:**
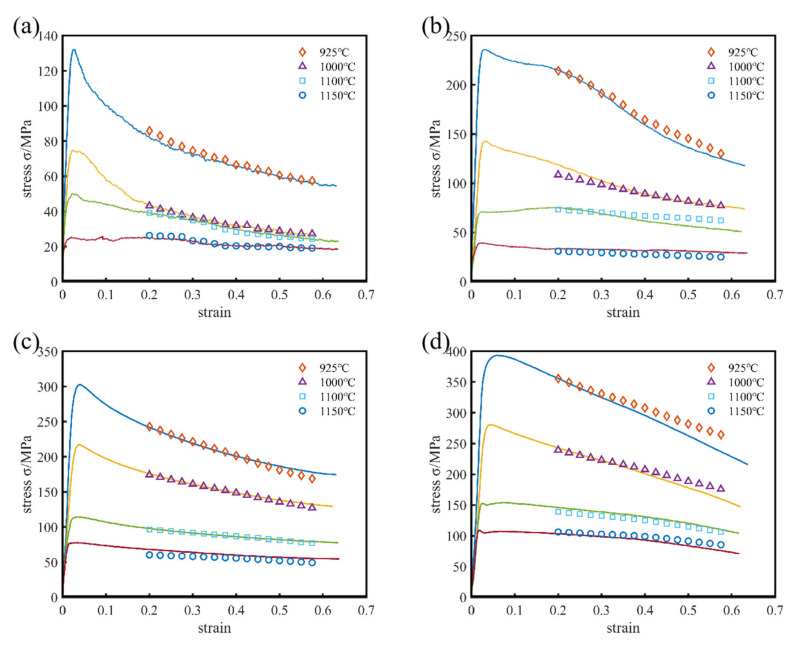
Comparison between the predicted and measured flow curves for Fe-15Mn-15Al-5Ni-1C low density steel at different strain rates: (**a**) 0.01 s^−1^, (**b**) 0.1 s^−1^, (**c**) 1.0 s^−1^, and (**d**) 10 s^−1^.

In order to further verify the accuracy of the constitutive equation model, the stresses obtained via hot compression were compared with the calculated stresses. A comparison between the predicted and measured values of the strain-compensated Arrhenius-type constitutive equation for this low-density steel experimental specimen under different hot deformation conditions is shown in Figure 8. The experimental stress values were very close to the predicted stress values.

**Figure 9:**
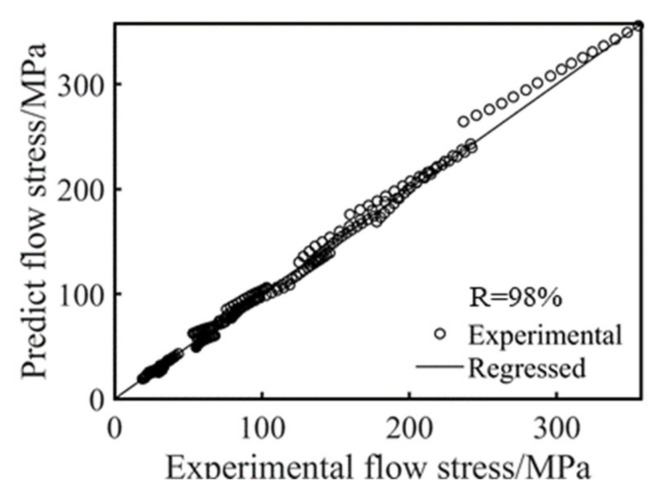
Comparison between the experimental and predicted flow stresses.

Figure 9 shows a comparison of the predicted and actual stress values. As can be seen from Figure 9, the correlation coefficient (R) between the experimental and predicted flow stress values was 98%. The experimental and predicted data for this range of heat deformation conditions had a high degree of agreement in the overall case, which meant that the constructed constitutive equation had good predictive capability.

### 3.4. Critical Conditions of DRX

Previous studies [26] defined the work-hardening rate, as given in Equation (11). The critical condition for DRX to occur in Fe-15Mn-15Al-5Ni-1C low-density steel was the inflection point of the θ–σ curve, which generally preceded the peak strain, with the critical condition satisfying Equation (12).

(11) $$\theta ={\left(\frac{d\sigma }{d\varepsilon }\right)}_{\dot{\varepsilon },T}$$

(12) $$\frac{\partial }{\partial \sigma }\left(-\frac{\partial \theta }{\partial \sigma }\right)=0$$

**Figure 10:**
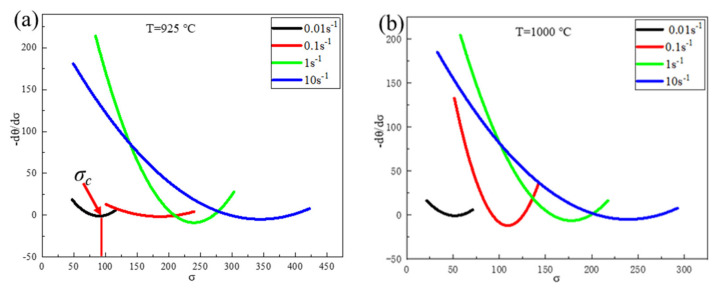

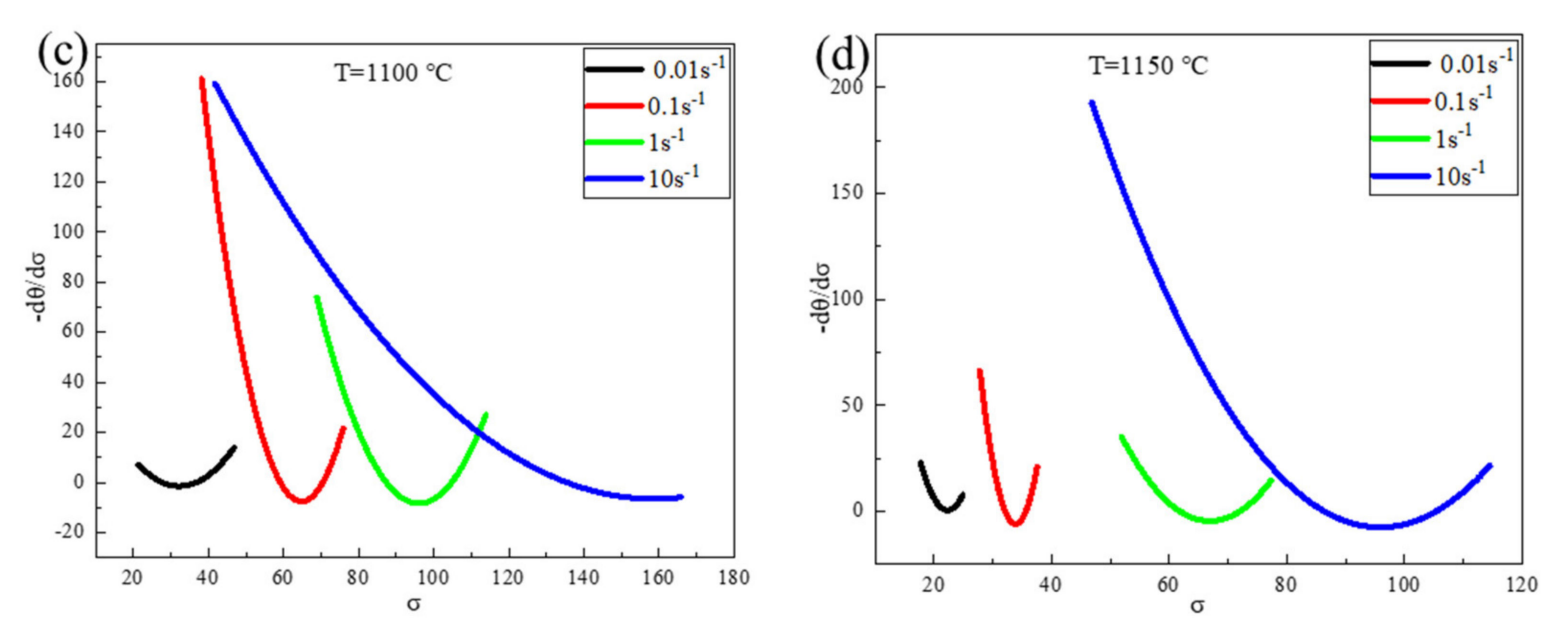
(–dθ/dσ)–σ curves of Fe-15Mn-15Al-5Ni-1C low-density steel under different deformation conditions: (**a**) 925 °C; (**b**) 1000 °C; (**c**) 1100 °C; (**d**) 1150 °C.

Figure 10 shows the σ–(–dθ/dσ) curves for the Fe-15Mn-15Al-5Ni-1C low-density steel under different deformation conditions. From Figure 10, it can be observed that there were minima in the σ–(–∂θ/∂σ) curves for different deformation conditions corresponding to the value of σ_c_ for DRX that occurred in the Fe-15Mn-15Al-5Ni-1C low-density steel. At the same deformation temperature, the critical stress value for DRX occurring in the Fe-15Mn-15Al-5Ni-1C low-density steel increased as the strain rate increased.

**Figure 11:**
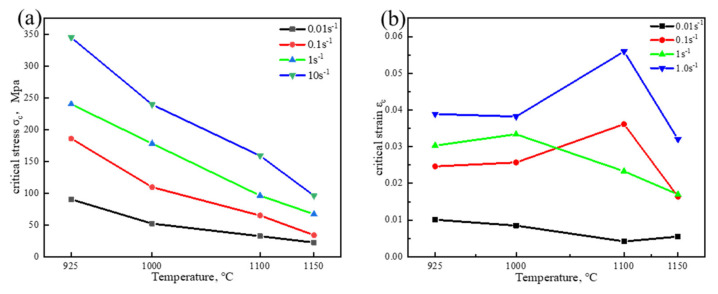
Critical condition versus deformation temperature curves of DRX for the Fe-15Mn-15Al-5Ni-1C low-density steel. (**a**) σ_c_–T curve; (**b**) ε_c_–T curve.

Based on the value of σ_c_ obtained in Figure 10, the critical strain value ε_c_ for DRX occurred in Fe-15Mn-15Al-5Ni-1C low-density steel could be obtained. The relationship curves of σ_c_–T and ε_c_–T were plotted under different deformation conditions, as shown in Figure 11. It was found in Figure 11 that both the hot deformation temperature T and the strain rate $\dot{\varepsilon }$ had a certain effect on the values of σ_c_ and ε_c_ of DRX in the Fe-15Mn-15Al-5Ni-1C low-density steel, which increased as the strain rate increased. An explanation for this was that when $\dot{\varepsilon }$ was low, although a large proliferation of dislocations within the crystal was caused by deformation, there was sufficient time for dislocation slipping such that the DRX nucleation and growth of austenite and ferrite were ensured, making it possible for DRX to occur when the σ and ε values were small. On the other hand, at a certain deformation temperature, the Fe-15Mn-15Al-5Ni-1C low-density steel deformed at a higher $\dot{\varepsilon }$, resulting in a larger value of θ. This was due to the fact that at the early stage of deformation, the dislocations inside the grains did not have enough time to slip and became entangled with each other, resulting in internal stress in the microstructure of the Fe-15Mn-15Al-5Ni-1C low-density steel, which required a larger deformation to provide more energy for the movement of dislocations to enable the Fe-15Mn-15Al-5Ni-1C low-density steel to undergo DRX. Therefore, a decrease in $\dot{\varepsilon }$ was favorable for the DRX behavior of the Fe-15Mn-15Al-5Ni-1C low-density steel. Under the same strain rate, with an increase in deformation temperature, the value of σ_c_ for DRX occurring in the Fe-15Mn-15Al-5Ni-1C low-density steel decreased. This was because a higher deformation temperature increased the diffusion migration of Al solute atoms such that the driving force for dislocations to undergo slip and creep movements was enhanced. At the same time, a higher deformation temperature led to enhanced grain boundary migration in the steel. Thus, at higher deformation temperature and lower flow stresses, DRX behavior occurred in this Fe-15Mn-15Al-5Ni-1C low-density steel. It is worth noting that the ε_c_ value of DRX for this Fe-15Mn-15Al-5Ni-1C low-density steel remained essentially constant with increasing deformation temperature at the same strain rate. The reason for this was that the process of DRX of this Fe-15Mn-15Al-5Ni-1C low-density steel started at the early stage of deformation and occurred before the peak stress. Due to strong work hardening, the flow stress reached a peak in a very short period, resulting in an essentially constant critical strain value at which recrystallization occurred.

The Sellars [27] model was introduced to characterize ε_c_, as shown in Equation (13):

(13) $$\varepsilon_{c}={\mathrm{aZ}}^{b}$$

where a and b are material constants and Z is the Zener–Hollomon parameter. Taking the logarithm of both sides of Equation (13) and then linearly regressing the fitted curve, the ε_c_ prediction model for the Fe-15Mn-15Al-5Ni-1C low-density steel is shown in Equation (14). According to Equation (5), a higher deformation temperature T led to a smaller Z value and a smaller ε_c_ value, thus promoting the DRX behavior of the Fe-15Mn-15Al-5Ni-1C low-density steel, while a faster $\dot{\varepsilon }$ led to a larger Z value and a larger ε_c_ value, thus inhibiting the DRX behavior of the Fe-15Mn-15Al-5Ni-1C low-density steel. The results of the fitting of the critical strain to peak strain relationship are shown in Equation (15). The DRX behavior of the steel had occurred before the hot compression deformation reached σ_p_.

(14) $$\varepsilon_{c}=7.48\times 10^{-5}Z^{0.1152}$$

(15) $$\varepsilon_{c}=0.7938\varepsilon_{p}-0.03301$$

### 3.5. Analysis of Microstructure Evolution

**Figure 12:**
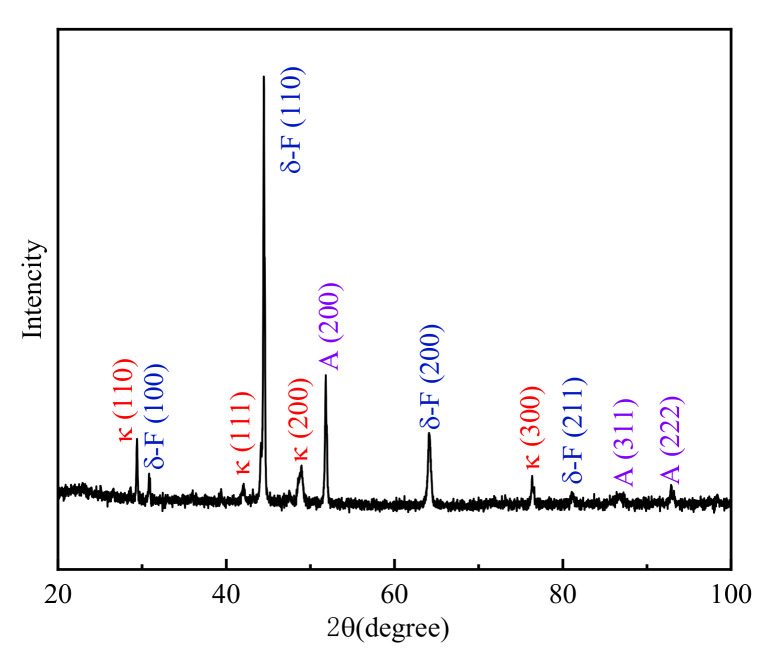
XRD spectrum of the Fe-15Mn-15Al-5Ni-1C steel after hot compression at T = 1150 °C and $\dot{\varepsilon }$ = 1.0 s^−1^.

Figure 12 shows the XRD pattern of this low-density steel at a deformation temperature of 1150 °C and a strain rate of 1.0 s^−1^. As shown in Figure 12, this low-density steel mainly contained δ-F, A, and κ carbides. 

**Figure 13:**
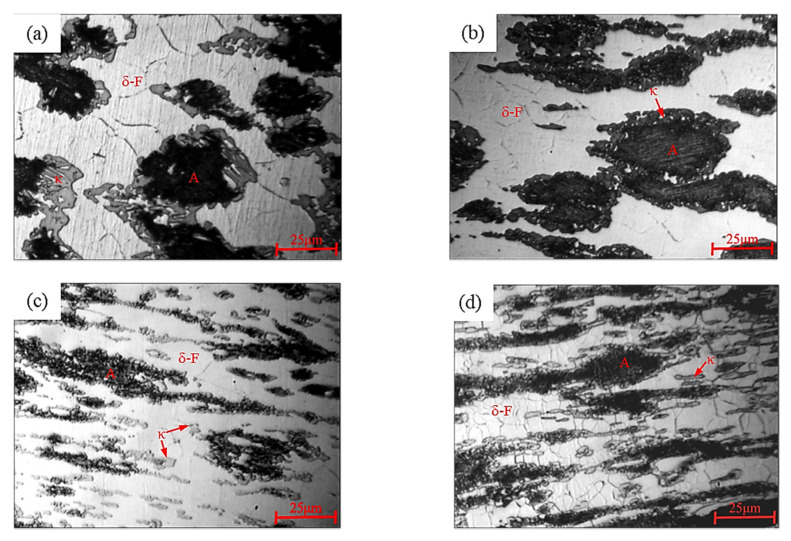
Microstructure of the Fe-15Mn-15Al-5Ni-1C low-density steel after hot compression at a temperature of 1100 °C: (**a**) $\dot{\varepsilon }$ = 0.01 s^−1^; (**b**) $\dot{\varepsilon }$ = 0.1 s^−1^; (**c**) $\dot{\varepsilon }$ = 1.0 s^−1^ and (**d**) $\dot{\varepsilon }$ = 10 s^−1^.

As shown in Figure 13, the steel consisted mainly of A and banded δ-F. At a deformation temperature of 1100 °C, δ-F caused DRX behavior, where the strain rate increased from 0.01 s^−1^ to 10 s^−1^ and the size of the δ-F grains decreased from 15 μm to 8 μm due to insufficient time for growth; meanwhile, the DRX behavior of A was not obvious and decomposed into α-ferrite (α-F) and κ carbides. As shown in Figure 13a, the δ-F grains were larger but more uniform in size under a 0.01 s^−1^ deformation condition. κ carbide nucleates grew at the A and δ-F phase boundary, resulting from a form of intergranular precipitation, where the strain rate was slow and the κ carbides had sufficient time to grow. As seen in Figure 13b, where the strain rate increased to 0.1 s^−1^, κ carbides also precipitated at the δ-F grain boundaries. In Figure 13c, for a strain rate of 1.0 s^−1^, the δ-F was banded, and due to the higher deformation temperature, the grain boundary of δ-F migrated, splitting A into island-like microstructures, with a large amount of κ carbides precipitating at the A and δ-F phase boundaries or δ-F grain boundaries. As shown in Figure 13d, when the strain rate increased to 10.0 s^−1^, the δ-F grains varied in size and morphology and appeared to be polygonal in character. Long stripes of κ carbides were present at the δ-F grain boundaries.

**Figure 14:**
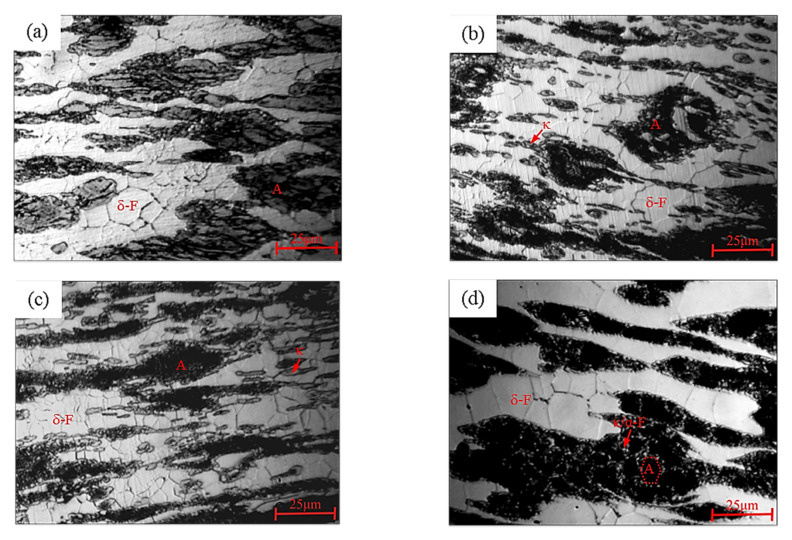
Microstructure of the Fe-15Mn-15Al-5Ni-1C low-density steel after hot compression at a strain rate of 10 s^−1^ for (**a**) T = 925 °C, (**b**) T = 1000 °C, (**c**) T = 1100 °C, and (**d**) T = 1150 °C.

Figure 14 shows the microstructural characteristics of the Fe-15Mn-15Al-5Ni-1C low-density steel at different deformation temperatures at a strain rate of 10 s^−1^. As shown in Figure 14, the microstructure of this Fe-15Mn-15Al-5Ni-1C low-density steel at this deformation condition was mainly composed of δ-F and A, with δ-F showing band-like distributions. As the deformation temperature increased from 925 °C to 1150 °C, the size of the δ-F DRX grains increased slightly. Due to the excessive strain rate, the δ-F grains were all smaller and the number of δ-F recrystallizations increased significantly, which were consistent with the changing characteristics of the stress–strain curve of this steel and were more conducive to the occurrence of DRX during high-temperature deformation, where dynamic softening occurred, lowering the deformation resistance [28]. α-F and κ carbides may have been present in zone A. As shown in Figure 14a, the deformation temperature was 925 °C at this point and A had recrystallized as elongated grains. As shown in Figure 14b, when the deformation temperature was 1000 °C, the distribution of κ carbides was more scattered, and most existed at the phase boundary between A and δ-F or the δ-F grain boundary. This was probably because the increase in temperature provided higher energy for the formation of κ carbides. As shown in Figure 14c, the deformation temperature at this point was 1100 °C. The distribution of κ carbides did not change much from that at 1000 °C, but the size and shape of δ-F had changed. As shown in Figure 14d, the deformation temperature was higher and a co-precipitation reaction occurred in zone A, where a mixture of chain-like α-F and κ carbides had precipitated.

**Figure 15:**
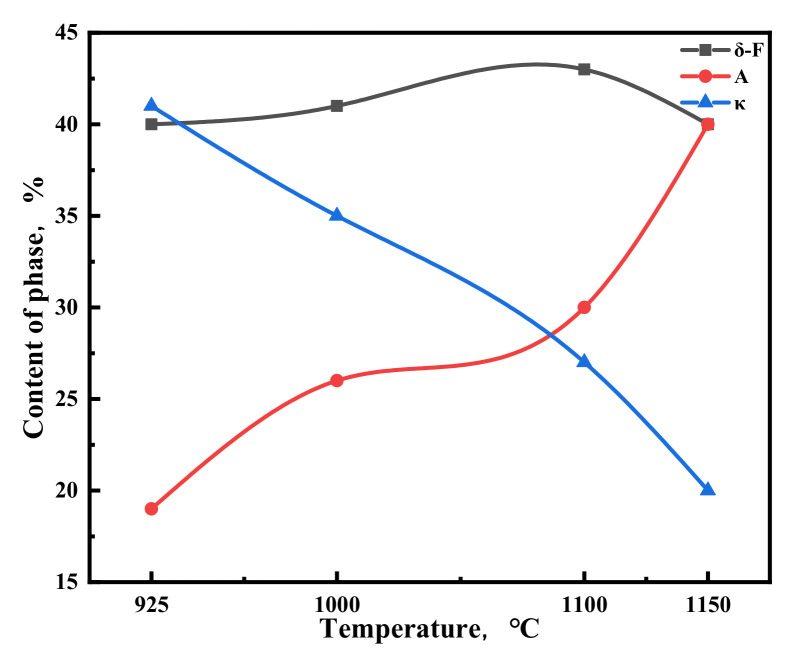
Contents of the phases in the microstructure of Fe-15Mn-15Al-5Ni-1C low-density steel after hot compression at a strain rate of 10 s^−1^.

Figure 15 shows the percentage of each phase in the microstructure of Fe-15Mn-15Al-5Ni-1C steel at different deformation temperatures at a strain rate of 10 s^−1^. As shown in Figure 15, excluding the absolute errors in local sampling, the hot deformation temperature has little effect on the δ-F content during the increase from 925 °C to 1150 °C. The opposite pattern of change in the content of A and κ carbides with the increase in hot deformation temperature occurred, which was due to the enhanced solid solution effect of Fe-15Mn-15Al-5Ni-1C steel with the increase in hot deformation temperature, where the κ carbides solid solution transformed into A, making the content of A increase, while the content of κ carbides decreased.

**Figure 16:**
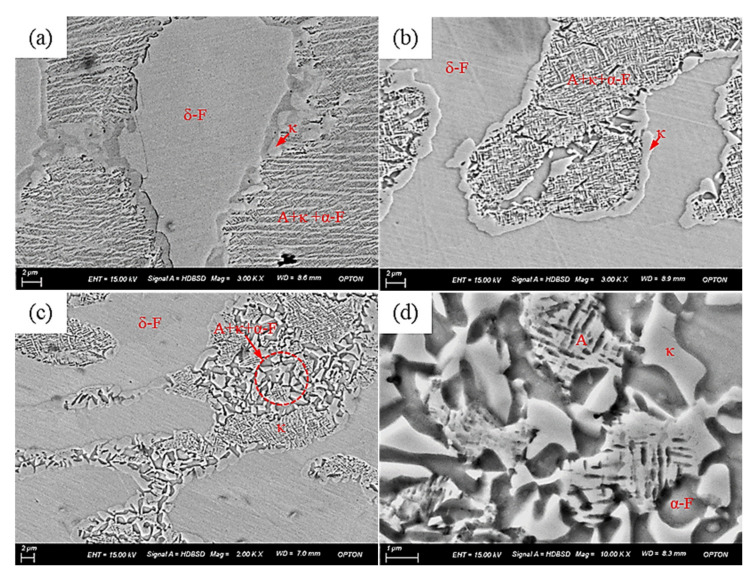
Microstructure of the Fe-15Mn-15Al-5Ni-1C low-density steel under different deformation conditions: (**a**) T = 1100 °C, $\dot{\varepsilon }$ = 0.1 s^−1^; (**b**) T = 1100 °C, $\dot{\varepsilon }$ = 10 s^−1^; (**c**) T = 1150 °C, $\dot{\varepsilon }$ = 1.0 s^−1^; (**d**) T = 1150 °C, $\dot{\varepsilon }$ = 10 s^−1^.

Figure 16 shows the microstructure of the Fe-15Mn-15Al-5Ni-1C low-density steel under different deformation conditions. It can be seen that the microstructure of the austenite zone of the steel varied at different deformation temperatures and different strain rates. Figure 16a shows the microstructure of the Fe-15Mn-15Al-5Ni-1C low-density steel at a deformation temperature of 1100 °C and a strain rate of 0.1 s^−1^; there were many κ carbides with sizes of about 1 μm to 2 μm that had precipitated at the phase boundary between δ-F and A, while the interior of A was uniformly distributed with many nanoscale κ carbides and elongated α-F, which resulted from the amplitude decomposition of A. Microbands also appeared in this region, which contributed to the plastic toughness and strength of the steel. Figure 16b shows the microstructure of the Fe-15Mn-15Al-5Ni-1C low-density steel at a deformation temperature of 1100 °C and a strain rate of 10 s^−1^. As shown in Figure 16b, continuous κ carbides precipitated at the phase boundary of δ-F and A. This was due to the diffusion of alloying elements, namely Al and Ni elements in δ-F and Mn and C elements in A, which diffused to the phase boundary of δ-F and A where they were enriched, leading to the formation of κ carbides; meanwhile, the higher energy out of the phase boundary also facilitated the formation of κ carbides. Figure 16c shows the microstructure of the Fe-15Mn-15Al-5Ni-1C low-density steel at a deformation temperature of 1150 °C and a strain rate of 1.0 s^−1^. It can be seen that there were two distinct regions in the A zone. During the cooling process after hot deformation, austenite bar decomposition occurred, which led to the diffusion of C and Al atoms, an increased difference in solute atom concentration, the formation of solute-poor and solute-rich zones, and ordered phases in the solute-rich zone precipitate that were uniformly distributed in the austenite, which eventually formed κ carbide. As shown in the red circles in Figure 16c, in the solute-poor zone, A decomposed to form α ferrites and κ carbides, eventually forming a three-phase zone of A, α ferrites, and κ carbides. Finally, Figure 16d shows the microstructure of the Fe-15Mn-15Al-5Ni-1C low-density steel at a deformation temperature of 1150 °C and a strain rate of 10 s^−1^. It can be seen that A decomposed into α-F, κ carbides, and residual austenite; this was also found in a study by Degang Liu [29] and others.

## 4. Conclusions

The hot deformation behavior of Fe-15Mn-15Al-5Ni-1C low-density steel in the as-cast state was investigated through the establishment of the constitutive equation and analysis of the microstructure, which provided reference significance for the TMP of Fe-15Mn-15Al-5Ni-1C low-density steel. The main conclusions of our study are summarized in the following.

1. The microstructure of Fe-15Mn-15Al-5Ni-1C low-density steel ingots was mainly composed of austenite and ferrite phases with a dendritic shape. The contents of two alloying elements, namely, Al and Ni, were higher in ferrite than that in austenite, while the contents of the other two alloying elements, namely, Mn and C, were lower in ferrite than that in austenite. The microstructure of the 15Al-5Ni-1C low-density steel after hot compression was mainly composed of δ-F, A, α-F, and κ carbides.
2. After hot compression deformation, the microstructure of the Fe-15Mn-15Al-5Ni-1C low-density steel was mainly composed of δ-F, A, α-F, and κ carbides. When the deformation temperature was below 1150 °C, κ carbides precipitated on the phase boundary between A and δ-F, or on the δ-F grain boundary; when the deformation temperature reached 1150 °C, κ carbides formed inside A via a co-precipitation reaction.
3. At higher strain rates ($\dot{\varepsilon }$ = 10 s^−1^), the Fe-15Mn-15Al-5Ni-1C low-density steel exhibited significant work hardening and subsequent rapid softening characteristics, while at lower strain rates ($\dot{\varepsilon }$ < 10 s^−1^), the flow behavior of the alloy was dominated by a steady-state flow. The Fe-15Mn-15Al-5Ni-1C low-density steel had an average activation energy of deformation of Q = 540 kJ mol^−1^, a work hardening index of n = 4, and its hot deformation mechanism was solute dragging and dislocation climbing controlled by the strain rate.
4. Under the same hot deformation temperature, with an increase in the strain rate, the critical stress value and critical strain value of DRX that occurred in the Fe-15Mn-15Al-5Ni-1C low-density steel increased. Under the same strain rate, with an increase in the hot deformation temperature, the critical stress value of DRX that occurred in the Fe-15Mn-15Al-5Ni-1C low-density steel decreased and the recrystallization grain size increased, where the grains were easily polygonized.

## Figures and Tables

**Table 1:** Chemical composition of the Fe-15Mn-15Al-5Ni-1C low-density steel (wt.%).

Mn	Al	Ni	C	Fe
15.0	15.0	5.0	1.0	Bal.

**Table 2:** Hot deformation activation energy of Fe-Mn-Al-C-based low-density steels.

Material	Deformation Temperature (°C)	Q	n	Phases Present
Fe-9Al-10.8Mn-4.5Ni-0.7C [6]	950–1150	400	4.08	A + F + κ + B2
Fe-11Mn-10Al-0.9C [8]	950–1100	356	3.4429	A + δ-F
Fe-27Mn-11.5Al-0.95C [21]	900–1150	294	3.93	A + δ-F
Fe-15Mn-15Al-5Ni-1C	925–1150	540	4	δ-F + A + α-F + κ

**Table 3:** Polynomial fitting results of α, n, Q, and lnA for the Fe-15Mn-15Al-5Ni-1C low-density steel experimental alloy.

α	n	Q	lnA
X_0_ = −1.260 × 10^−3^	Y_0_ = 2.172 × 10^1^	Z_0_ = 1.558 × 10^4^	E_0_ = 1.424 × 10^2^
X_1_ = 1.809 × 10^−1^	Y_1_ = −3.272 × 10^2^	Z_1_ = −1.865 × 10^5^	E_1_ = −1.737 × 10^3^
X_2_ = −1.123	Y_2_ = 2.340 × 10^4^	Z_2_ = 1.293 × 10^6^	E_2_ = 1.203 × 10^4^
X_3_ = 3.779	Y_3_ = −8.634 × 10^4^	Z_3_ = −4.694 × 10^6^	E_3_ = −4.361 × 10^4^
X_4_ = −6.907	Y_4_ = 1.730 × 10^5^	Z_4_ = 9.242 × 10^6^	E_4_ = 8.582 × 10^4^
X_5_ = 6.499	Y_5_ = −1.789 × 10^5^	Z_5_ = −9.375 × 10^6^	E_5_ = −8.705 × 10^4^

## Data Availability

Not applicable.
